# The mediating role of attitudes to aging in the relationship between information overload, cyberchondria, and influenza vaccine hesitancy among Chinese older adults

**DOI:** 10.3389/fpubh.2026.1743367

**Published:** 2026-04-16

**Authors:** Cheng Cheng

**Affiliations:** College of Communication Arts and Sciences, Beijing Information Science and Technology University, Beijing, China

**Keywords:** cyberchondria, influenza vaccine hesitancy, information overload, self-perceptions of aging, stereotype embodiment theory

## Abstract

**Background:**

In the context of a rapidly aging global population, influenza vaccine hesitancy among Chinese older adults remains a critical public health issue. Concurrently, the proliferation of digital health information has raised concerns about the negative effects of information overload and cyberchondria. However, the psychological pathways linking these digital phenomena to vaccine hesitancy, particularly for older adults, remain underexplored.

**Objectives:**

This study aims to examine the association between health information overload on excessive online search (cyberchondria) and influenza vaccine hesitancy among Chinese older internet users. Grounded in the Stereotype Embodiment Theory (SET), the study specifically examines the mediating role of self-perceptions of aging.

**Methods:**

A self-reported online questionnaire was distributed to 791 Chinese internet users aged 55 or older. The research hypotheses were tested using Partial Least Squares Structural Equation Modeling (PLS-SEM) in SmartPLS.

**Results:**

Results indicate that health information overload was significantly correlated with cyberchondria (β = 0.501, *p* < 0.001), psychological growth (β = −0.236, *p* < 0.001), physical change (β = −0.229, *p* < 0.001), and psychosocial loss (β = 0.254, *p* < 0.001). Similarly, cyberchondria was a significant predictor of psychological growth (β = −0.184, *p* < 0.001), physical change (β = −0.312, *p* < 0.001), and psychosocial loss (β = 0.285, *p* < 0.001). Finally, physical change (β = −0.209, *p* < 0.001) and psychosocial loss (β = 0.247, *p* < 0.001) showed a significant statistical relationship with vaccine hesitancy.

**Conclusions:**

The findings highlight the critical importance of fostering positive aging attitudes in health communication strategies. Health practitioners and policymakers should develop age-friendly digital environments and educational campaigns that not only provide accurate information but also empower older adults to perceive aging more rationally, thereby potentially encouraging proactive health behaviors.

## 1 Introduction

The burgeoning aging population over the past few decades has underscored the imperative for controlling seasonal influenza, given that approximately 90% of flu-related deaths occur among high-risk adults aged 60 and above (1). In light of this, the World Health Organization (WHO) recommends that older adults, especially those with underlying chronic conditions, receive annual vaccination due to their heightened susceptibility to severe outcomes from seasonal influenza infections (2).

In China, the expanding older population, coupled with the recurrent burden of influenza epidemics, has significantly increased the demand for health resources, particularly for health education and vaccination (3). While influenza-related illness, complications, and deaths are preventable via vaccination programs, there is an urgent need to address the consistently low vaccination acceptance rates among the older population, which are driven in part by vaccine hesitancy (4). This is underscored by official statistics, which showed that during the 2022–2023 epidemic season, only 3.8% of individuals aged 60 and over received an influenza vaccination (5). Vaccine hesitation is defined as an individual's skepticism or doubt about vaccine safety and efficacy (6). Previous research has identified that digital media use, socio-demographic status, psychological factors, and health perceptions are the crucial antecedents of older adults' vaccination acceptance (7, 8).

Recently, a growing body of literature has focused on the wide-ranging effects of digital media on older adults' health outcomes. This is particularly relevant in China, where, according to the China Internet Network Information Center, by December 2025, the number of internet users aged 60 and above in China reached 161 million. However, variations in digital literacy, specifically the capacity to critically navigate and verify complex health information, have led to contradictory findings regarding whether increasing reliance on communication technologies and more accessible health information sources exactly help them to make informed health decisions (9, 10).

Accordingly, the proliferation of digital health information across various platforms raises concerns about the impact of infodemic-driven misunderstanding and overuse of online health search among Chinese older adults (11). In this context, health information overload refers to a situation in which individuals are unable to process and distinguish a flood of information inputs, particularly when reliable news is mixed with false information (12, 13). Additionally, cyberchondria is defined as the phenomenon of excessive online searching for health information, further leading to panic, confusion, and worse health outcomes (14, 15). On the one hand, older adults, due to their pressing health concerns and weaker digital literacy skills, are more susceptible to uncritically scanning and compulsively seeking health-related information online (16). On the other hand, empirical evidence shows that daily exposure to a vast amount of always-accessible health information, which often surpasses an individual's processing capacity, can lead to both cyberchondria and health misperceptions, ultimately undermining vaccination intentions (17–19).

Beyond digital media use, studies have documented that the adoption of preventive behaviors is also mediated by individuals' health beliefs, including their attitudes toward aging. Existing research has shown that individuals' health-related attitudes and professional decisions are often shaped by complex psychological mechanisms, such as risk perception and negative emotional responses (20). Within the context of older adults, self-perceptions of aging (SPA) refer to an individual's subjective evaluations of age-related growth and decline across three dimensions: physical, psychological and social (21, 22). At some point, this multidimensional construct reflects how older adults understand and interpret their own aging process (23). On the one hand, the continuous exposure to negative health information and ageist content on digital platforms may directly shape how older adults perceive their own aging process (24). On the other hand, extensive research has demonstrated that attitudes toward aging (or self-perceptions of aging) are key contributors to older adults' cognitive performance, behavioral tendencies, and wellbeing across the lifespan (25, 26). While the Stereotypes Embodiment Theory (SET) focuses on the internalization of aging stereotypes, empirical extensions of this framework have increasingly recognized subjective age as a critical psychological factor that moderates how older adults process age-related cues and make health decisions (27).

In conclusion, although extensive research has revealed the determinants and health effects of older adults' self-perceptions of aging and has shown that their age-related thoughts can significantly influence health outcomes, few studies have demonstrated the link between infodemic and these perceptions. Although existing research has consistently shown that information overload and cyberchondria have a detrimental effect on preventive behaviors by influencing individuals' health perceptions, the mediating role of self-perceptions of aging remains unclear. Last but not least, there is an urgent need to elucidate the psychological mechanisms connecting information overload and cyberchondria to attitudes toward aging and, ultimately, to influenza vaccine hesitancy. This is particularly crucial for older adults, who are not only a vulnerable population in the digital age but also a primary focus for influenza control.

Hence, the primary objective of this study is to investigate the psychological pathways through which information overload and cyberchondria relate to influenza vaccine hesitancy among Chinese older adults. More specifically, this study examines whether and how self-perceptions of aging serve as a mediator in the relationships between these digital media-related factors and influenza vaccine hesitancy. By identifying this psychological pathway, our findings will not only advance the theoretical understanding of health communication in the digital age but also offer actionable insights for public health authorities to design targeted interventions aimed at mitigating influenza vaccine hesitancy among older adults.

## 2 Theoretical framework and hypotheses development

The stereotypes embodiment theory (SET) posits that individuals' attitudes toward aging have been shaped throughout life unconsciously. In later life, the collection of judgments that older adults hold toward their aging-related changes intentionally or unintentionally impacts their health status (21, 28). These optimistic and pessimistic perceptions of aging are dependent on a variety of factors, including personal experiences, intergenerational support, and social norms (29, 30).

Consistent with SET, internalized aging stereotypes are embodied and enacted in three pathways: psychological, behavioral, and physiological (24). Accordingly, past studies have categorized the consequences of an individual's attitudes toward aging into three subscales: (a) Psychological growth (PG): referring to positive gains about aging, such as wisdom, privilege; (b) Physical change (PC): representing positive expectations toward maintaining physical health; and (c) Psychosocial loss (PL): which involves a negative evaluation of social loss (31, 32).

Thus far, several studies have identified a correlation between self-perceptions of aging and health outcomes. It has been demonstrated that optimistic attitudes toward aging may collectively contribute to better physical and mental health among older adults. Conversely, their negative perceptions have adverse and accumulative impacts on self-efficacy beliefs, psychological status and even the will to live (23, 33). At a behavioral level, research consistently shows that older adults' positive views of aging are predictive of active engagement in health behaviors, such as nutrition, exercise and other preventive behaviors, potentially leading to a better quality of life (34, 35).

Extensive research has enriched our understanding that perceived information overload exerts a negative influence on psychological states and behavioral intentions of internet users (19, 36). Furthermore, it has been noted that the difficulties involved in handling digital health information make the older adults aware of being behind the times, thereby resulting in obsessive online searching, negative emotional reactions, and lower motivations to engage in health behaviors (37, 38).

**Figure 1:**
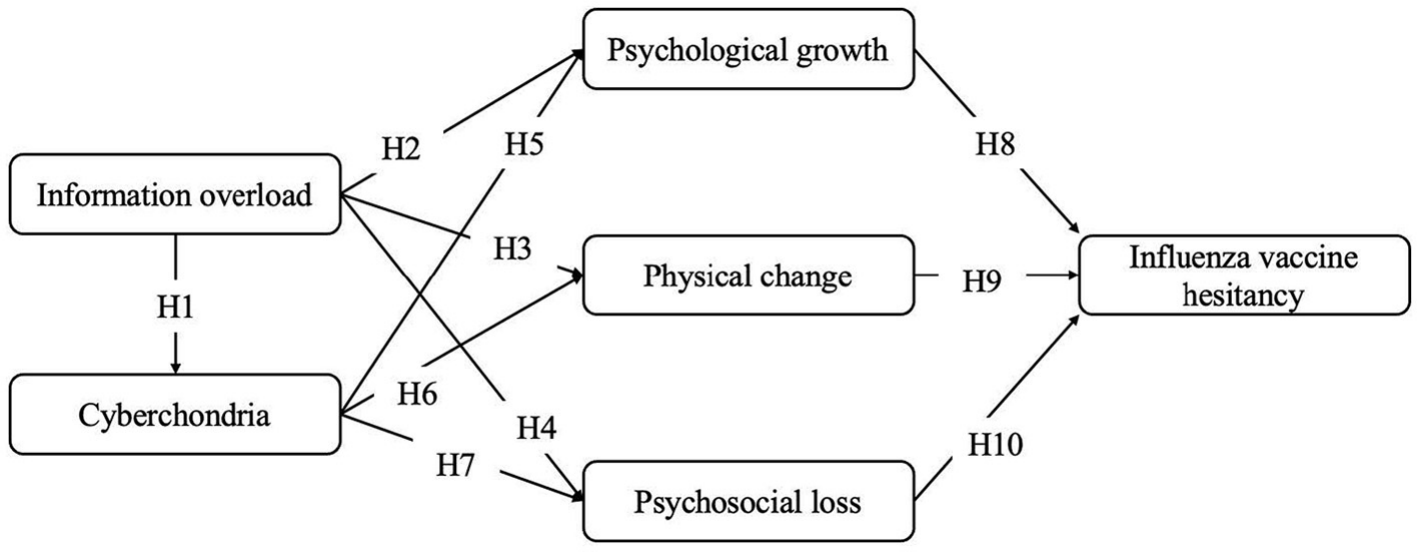
Theoretical framework.

Taken together, the research model and hypotheses were proposed as follows (see Figure 1):

H1: Health information overload is positively associated with perceived cyberchondria.

H2&H3: Health information overload is negatively associated with older adults' psychological growth and physical change.

H4: Health information overload is positively associated with psychosocial loss.

H5&H6: Perceived cyberchondria is negatively associated with older adults' psychological growth and physical change.

H7: Perceived cyberchondria is positively associated with older adults' psychosocial loss.

H8: Psychological growth is negatively associated with influenza vaccine hesitancy.

H9: Physical change is negatively associated with influenza vaccine hesitancy.

H10: Psychosocial loss is positively associated with influenza vaccine hesitancy.

## 3 Methods

### 3.1 Data collection

To achieve the aforementioned objectives, a self-reported questionnaire was distributed via the Credamo platform between March to August 2025. Criteria for selecting the participants were as follows: (1) Chinese adults aged 55 years or older; (2) regular internet users; (3) able to comprehend and complete the questionnaire; and (4) did not receive the influenza vaccine. A total of 791 participants who met these criteria were included in the final analysis. Written informed consent was obtained from all participants, ensuring they voluntarily participated and understood the purpose of the research and data processing procedures. Ethical approval for this study was obtained from the Ethics Committee of the authors' affiliated university.

### 3.2 Measurement

The socio-demographic characteristics, including age, gender, education, and monthly income, were collected from all participants.

Perceived cyberchondria was measured using a three-item scale adapted from previous validation studies (15, 16). Participants rated the extent to which they searched excessive web pages about the same medical condition and felt distressed or nervous after researching online. Responses were measured on a five-point Likert scale, ranging from 1 (Never) to 5 (Always).

In terms of health information overload, participants were asked to rate the degree to which they felt burdened when handling online health information, using a five-point scale from 1 (Strongly Disagree) to 5 (Strongly Agree) (36, 37).

Self-perceptions of aging have been measured from two perspectives: one is to identify the person's subjective age (also known as “felt age”). The participants were asked, “How old do you feel?” (25). The other is to evaluate participants' self-perceptions of age-related gains and losses based on the shortened format of the Attitudes to Aging Questionnaire (AAQ), which has been widely validated across different cultural contexts (39). This study adopted three key subscales: Psychological Growth (PG), Physical Change (PC), and Psychosocial Loss (PL), each containing at least three items. The adapted scale was specifically tailored for Chinese older adults (31). All items were rated on a five-point Likert scale from 1 (Strongly Disagree) to 5 (Strongly Agree).

Regarding influenza vaccine hesitancy, participants were asked to what extent they would be reluctant to follow the influenza vaccination plan (19). Responses were collected on a five-point Likert scale.

### 3.3 Statistical analysis

Initially, descriptive statistics were calculated for all demographic variables. A Kolmogorov-Smirnov test revealed the non-normal distribution of the data (*p* < 0.001). Subsequently, the proposed theoretical framework was tested using Partial Least Squares Structural Equation Modeling (PLS-SEM) via Smart PLS 4.1.1.2. PLS-SEM was chosen due to its suitability for exploratory research and its ability to handle complex models with both formative and reflective constructs.

The analysis proceeded in two steps. First, the measurement model was assessed for validity and reliability, including internal consistency reliability (Cronbach's alpha), convergent validity (AVE), and discriminant validity (Fornell-Larcker criterion). Second, the structural model was evaluated. The significance of path coefficients was determined using a complete bootstrapping procedure with 5,000 resamples. A *p*-value < 0.05 was considered statistically significant. Finally, the mediating effects of the SPA subscales were examined using the bootstrapping method.

## 4 Results

### 4.1 Descriptive analysis

A total of 791 older adults completed the survey. The overall sample was composed of internet users aged 55–60 years (60.4%), 60–70 years (33.1%), or over 70 years (6.4%). The majority of the sample was male (69.8%, *n* = 552). 34.0% of participants held a bachelor's degree or higher, and 66.9% reported a monthly income of more than 8,000 yuan (approximately $1100). 60.9% of participants reported they feel older than their actual age. Table 1 provides a comprehensive summary of all demographic data.

**Table 1:** Demographic characteristics (*N* = 791).

Factors		*N* (%)
Gender	Male	552 (69.8%)
	Female	239 (30.2%)
Age	55–60	478 (60.4%)
	60–70	262 (33.1%)
	over 70 years	51 (6.4%)
Income	Lower than 8,000	262 (33.1%)
	Higher than 8,000	529 (66.9%)
Education	High school or less	522 (66.0%)
	College degrees or higher	269 (34.0%)
Subjective age	Younger than chronological age or the same age	309 (39.1%)
	Older than chronological age	482 (60.9%)

### 4.2 Measurement model

To assess common method bias (CMB), Harman's single-factor test was performed. The first factor explained 33.530% of the total variance, well below the 50% threshold. Additionally, the KMO value was 0.891, and Bartlett's test was significant (*X*^2^ = 4,949.795, *df* = 153, *p* < 0.001).

The validity (convergent and discriminant validity) and reliability (indicator and internal consistency reliability) of the measurement model were assessed. Table 2 presents the results of these analyses.

**Table 2:** Assessment of the measurement model.

Constructs and item wording	Loading	VIF
Health information overload (IO) (Mean: 3.767; SD:0.942) (CR:0.818; AVE:0.600)		
I feel there is too much health information on the internet, exceeding my capacity to handle it	0.787	1.371
The excessive amount of online health information makes me feel burdened in processing and distinguishing it.	0.715	1.212
I have received a large supply of information, resulting in powerless and nervousness.	0.819	1.370
Cyberchondria (CY) (Mean: 3.340; SD:0.780) (CR: 0.835; AVE:0.752)		
I have researched excessive web pages about the same medical condition	0.872	1.945
I feel more panic or distressed after searching for symptoms repeatedly	0.882	2.184
I can't stop entering the same symptoms into different web pages that interrupted my offline activities.	0.847	1.816
Psychological growth (PG) (Mean: 2.722; SD:0.861) (CR: 0.746; AVE: 0.663)		
I have greater faith in my wisdom and experience	0.813	1.401
As people get older, they have more pleasant things	0.810	1.561
Aging is a kind of force and privilege	0.820	1.544
Physical change (PC) (Mean: 2.172; SD:0.690) (CR:0.734; AVE: 0.652)		
I am more active than I expected	0.811	1.450
I feel healthier than I expected	0.811	1.468
I don't feel old	0.801	1.432
Psychosocial loss (PL) (Mean: 3.263; SD:0.757) (CR:0.805; AVE: 0.629)		
As I get older, I find it more difficult to communicate with people around me	0.785	1.475
I think I can't fit into society, or can't make new friends	0.815	1.740
I feel like I am losing my physical independence	0.795	1.763
Influenza vaccine hesitancy (IV) (Mean: 3.307; SD:0.974) (CR: 0.708; AVE:0.774)		
I still won't follow the influenza vaccination plan in the future	0.869	1.430
I remain doubtful that the safety and effectiveness of influenza vaccination	0.890	1.430

Indicator reliability was confirmed as all factor loadings exceeded the recommended threshold of 0.7. Internal consistency reliability was verified by composite reliability (CR) values, all of which were above the acceptable threshold of 0.7. Convergent validity was established as the average variance extracted (AVE) values for all constructs were greater than the 0.5 threshold. Additionally, the variance inflation factors (VIF) for all constructs were below the recommended value of 5, indicating that multicollinearity was not a concern (40, 41). Discriminant validity was further confirmed using the Fornell-Larker criterion, as the square root of the AVE for each construct in the model satisfied the recommended threshold values based on the Fornell-Larker criterion, as shown in Table 3 (42). The results confirmed that the measurement model was reliable and valid, providing a strong foundation for testing the hypotheses.

**Table 3:** Discriminant validity (Fornell-Larcker criterion).

Constructs	IV	PC	CY	PG	IO	PL
**IV**	0.880					
**PC**	−0.341	0.808				
**CY**	0.355	−0.426	0.867			
**PG**	−0.191	0.392	−0.302	0.814		
**IO**	0.318	−0.385	0.501	−0.328	0.775	
**PL**	0.358	−0.547	0.413	−0.472	0.397	0.793

### 4.3 Hypothesis testing

Overall, the model explained 15.8% of the variance in influenza vaccine hesitancy among Chinese older adults. Table 4 presents the standardized path coefficients and their significance. Results showed a positive association between information overload and cyberchondria (β = 0.501, *p* < 0.001). H1 was supported.

**Table 4:** Results of hypothesis testing.

Hypothesis	Relationship	β	*T*-value
**H1**	IO and CY	0.501^**^	16.604
**H2**	IO and PG	−0.236^**^	5.773
**H3**	IO and PC	−0.229^**^	6.103
**H4**	IO and PL	0.254^**^	6.802
**H5**	CY and PG	−0.184^**^	4.580
**H6**	CY and PC	−0.312^**^	8.781
**H7**	CY and PL	0.285^**^	8.330
**H8**	PG and IV	0.007	0.186
**H9**	PC and IV	−0.209^**^	4.969
**H10**	PL and IV	0.247^**^	5.918

^**^p < 0.001.

As hypothesized, health information overload was found to be significantly and negatively correlated with older adults' psychological growth (β = −0.236, *p* < 0.001) and physical change (β = −0.229, *p* < 0.001), supporting hypotheses 2 and 3. Furthermore, health information overload showed a significant positive relationship with psychosocial loss (β = 0.254, *p* < 0.001), supporting hypothesis 4.

As postulated, cyberchondria was a significant predictor of older adults' influenza vaccine hesitancy through its statistical links with psychological growth (β = −0.184, *p* < 0.001), physical change (β = −0.312, *p* < 0.001), and psychosocial loss (β = 0.285, *p* < 0.001). Thus, H5, H6, and H7 were supported.

Regarding the associations between SPA and influenza vaccine hesitancy, two of the three dimensions were found to be statistically significant. Physical change was significantly and negatively associated with vaccine hesitancy (β = −0.209, *p* < 0.001), and psychosocial loss exerts a positive influence (β = 0.247, *p* < 0.001). These findings support H9 and H10. Contrary to our expectations, there was no statistical association between psychological growth and influenza vaccine hesitancy, rejecting hypothesis 8.

Other than the above, the estimated indirect association of health information overload on vaccination hesitancy is significant (β = 0.176, *p* < 0.001). Test of indirect relationships also found a significant positive correlation between cyberchondria and vaccination hesitancy among older adults (β = 0.134, *p* < 0.001).

### 4.4 *Post-hoc* analysis

Despite extensive research on self-perceptions of aging, the potential moderating role of subjective age remains an under-explored area. To address this, we conducted a *post-hoc* analysis using a multi-group approach to test whether felt age differentiates the proposed relationships. Participants were divided into two groups based on their self-reported subjective age: those who felt older than their chronological age (felt-older group) and those who felt younger or the same age (felt-younger group).

Bootstrap multigroup analysis revealed that the total association between health information overload on physical change was significantly stronger among participants in the felt-older group [β = −0.462, 95% CI: (−0.538, −0.370)] compared to the felt-younger group [β = −0.337, 95% CI: (−0.417, −0.247)] (diff. = 0.125, *p* < 0.05).

Similarly, a statistically significant difference was evident in the total relationship between information overload on older adults' perceptions regarding psychosocial loss, with a stronger effect among the felt-older group [β = 0.489, 95% CI: (0.388, 0.570)] compared to the felt-younger group [β = 0.336, 95% CI: (0.241, 0.420)] (diff. = 0.153, *p* < 0.05). None of the remaining differences were found.

## 5 Discussion

The current study investigates the association between health information overload, cyberchondria, and influenza vaccine hesitancy, as well as the potential role of older adults' subjective perceptions of aging. Our findings provide empirical insights into the proposed framework, indicating that the stereotypes embodiment theory (SET) offers a novel perspective for linking attitudes to aging and preventive health behaviors. However, it is important to note that the structural model demonstrates a modest explanatory power regarding influenza vaccine hesitancy. While this indicates that the SET framework captures critical psychological precursors, it also suggests that vaccine hesitancy is a multifaceted phenomenon significantly influenced by broader factors not fully captured in the current model, such as vaccine accessibility and social norms.

**The statistical associations between information overload and cyberchondria**

This study validates the link between health information overload and increased cyberchondria, negative views on aging, and influenza vaccine hesitancy. Similarly, cyberchondria was shown to be negatively associated with positive attitudes toward aging and positively related to vaccine hesitancy. This also accords with our earlier observations, which suggest that continuous exposure to both verified and unverified health information in the digital environment leads to information overload, increasing the risk of an excessive pattern of online searchers and misleading health decisions (37, 43). Notably, although both information overload and cyberchondria are significant predictors, our results also suggest they represent only a portion of the complex decision-making process, which may also be shaped by contextual factors, such as vaccine availability and interpersonal trust.

There are several possible explanations for these results. Firstly, consistent with the literature, a steady stream of health-related content from various online sources may be related to distinct functional impairment, thereby linking to negative emotional state, especially for older adults (11, 38). Secondly, faced with the repetition of negative online messages, older adults may become more aware of signs of age discrimination, subsequently interpreting these messages as self-relevant and perceiving physical aging negatively (44). Thirdly, in addition to these negative thoughts, older adults are easily misled by conflicting or false information, which in turn is associated with changes in their fragile social relationships (45). For instance, while clinical reports on rare post-vaccination complications provide valuable medical insights, they are often misinterpreted by laypeople as evidence of danger (46). Additionally, while digital environments can foster social relationships by facilitating interaction, our findings suggest that information overload and cyberchondria may exert an opposing pressure, exacerbating feelings of psychosocial loss (56).

Indeed, the internalization and embodiment of an individual's self-perceptions of aging are often more complex, as they are related to multifaceted factors such as societal structures and cultural stereotypes. For this reason, it is worth noting that older adults' negative perceptions of the aging process may be exacerbated not only by information redundancy and compulsive online searching but also by the excessive social comparison that occurs on digital platforms. Previous research has found that older adults often compare themselves to actual or imagined others regarding physical changes, appearance, and social status. While this has proven to be an effective method for persuading them to adopt preventive behaviors, it also poses a serious threat to healthy aging (47). Specifically, some studies have suggested that older people, especially female older adults, are more likely to evaluate their life and self-worth based on the posts shared by others who not only maintain physical health but also maintain a youthful appearance (48). However, as older adults are frequently exposed to a variety of selfie images with beautification filters and exaggerated identities of other people from multiple sources, their preoccupation could contribute to appearance concerns and unrealistic expectations of age-related changes (49).

Based on these findings, several strategies can be implemented to mitigate the negative effects of information overload and cyberchondria on older adults' self-perceptions of aging and vaccine hesitancy.

First, it is crucial for health communicators to realize the fact that while older adults need more vaccine knowledge, they are also more likely to disseminate and follow erroneous information than younger generations (9, 11). Hence, it is essential to seek a mindful balance for the older adults in obtaining, selecting, and distinguishing health information, considering their perceived social inferiority, memory issues, limited rumor-discrimination ability, and cognitive decline.

Second, informational support should focus on providing accurate health content that clarifies the effectiveness and safety of annual influenza vaccination, especially by addressing potential adverse reactions in older adults with chronic diseases (3). Health communicators should also consider individual differences in age-related experiences and health-related characteristics, as older adults often rely on a message's tone, popularity cues, and prior knowledge to judge credibility.

Third, apart from providing informational support, health communicators should recognize the significant role of emotional factors in older adults' vaccination decisions. The use of affective appeals, such as encouragement and understanding, is more effective and immediate than fear appeals in vaccination communication campaigns (50, 51). Finally, older adults should be reminded to rely on certified specialists and traditional media, rather than quick access to health-related content offered by laypeople on social media platforms.

**The mediating roles of self-perceptions of aging**

A key finding of this study is that the indirect associations of both information overload and cyberchondria on influenza vaccine hesitancy were statistically linked to two specific dimensions of self-perceptions of aging: physical change and psychosocial loss. This finding is consistent with broader empirical evidence suggesting that complex health decisions are often mediated by internal psychological mechanisms (20). While these mediators offer meaningful pathways, the unexplained variance highlights that older adults' vaccination intentions are also shaped by external socio-cultural structures beyond individual perceptions.

These findings broadly support the analysis of other studies indicating that the older adults who perceived their physical and cognitive changes positively, or experienced less psychosocial loss, are more likely to adopt health preventive behaviors (31, 33). A possible explanation for this is that older adults show more active engagement in collective movements and prosocial behaviors due to their higher demand for health self-management and social belonging (52, 53). Notably, our results revealed that psychological growth did not act as a significant mediator in this framework. This lack of significance suggests that while psychological growth represents internal wisdom and maturity, it may be less coupled with the immediate motivations required for preventive medical actions.

Consistent with this view, the finding that negative perceptions of health aging predict vaccination acceptance also supports age-based prejudices as a risk factor. Therefore, specific interpretations of aging in Chinese society, historical anecdotes, and ageism must be considered. Given that older adults may integrate age-related discriminatory information into their views on aging when making health-related decisions, health communicators need to foster a respectful environment for older adults (28). Since the mediating effect was primarily driven by physical and loss-related concerns, health communicators should leverage the interactive functions of the internet in vaccination education to provide peer support and alleviate social and emotional loneliness for older adults. As the views of older people are often shaped by younger generations' online opinions, a growing number of empirical studies reveal that promoting aging education targeting both young people and older adults via digital platforms may be a viable option for reducing ageism and combating health information overload, which is in turn linked to active participation in health behaviors (54).

Notably, this study found that subjective age significantly moderated the relationship between health information overload influenza vaccine hesitancy. Our results are consistent with previous studies, indicating that the associations between information overload and physical changes, as well as perceived psychosocial loss was stronger among participants with an older subjective age (25, 55). And such moderating roles of individual psychological traits are supported by recent research, which demonstrates how psychological capital serves as crucial construct in health behavioral models (20). While feeling younger is correlated with more active aging, these results must be interpreted with caution. This is because subjective age has been considered as a short-term variable that can fluctuate on a daily basis (27).

## 6 Conclusion

This research confirms previous findings surrounding the negative impact of infodemic-driven overuse of digital health information. Our findings lend crucial empirical support to the mediating role of self-perceptions of aging, demonstrating how the digital environment shapes older adults' attitudes toward aging, which in turn influences their vaccination decisions. This study reinforces the idea that vaccine hesitancy is a multi-dimensional phenomenon, with its drivers varying across age groups, digital technologies, and vaccine types. Furthermore, our findings are particularly salient in the Chinese context, where a unique combination of rapid digital technology adoption, traditional health beliefs, and a distinct public health system may uniquely shape the psychological pathways to vaccine hesitancy.

The results underscore the dual nature of the digital environment. On the one hand, the abundance of online health information can be beneficial, allowing older people to improve cognition, remain socially active, and attain successful aging. Conversely, these platforms can also expose older adults to false information, with all these difficulties directly or indirectly influencing their perceptions and health outcomes.

The empirical findings of this study highlight the considerable importance of addressing the negative aspects of older adults' self-perceptions of aging in addressing vaccine hesitancy. These findings are applicable to other health campaigns, such as promoting healthy lifestyle behaviors and chronic disease management. To this end, health communicators should foster supportive environments. For example, at the family and community levels, digital literacy should be enhanced via digital backfeeding, which refers to a form of intergenerational support focusing on three dimensions: improving access, building functional literacy, and mastering navigation skills. At the national and platform levels, stricter regulations are needed to manage health rumors and ensure the credibility of online information. Simultaneously, the government should strengthen public health communication by promoting positive aging narratives. These campaigns should specifically focus on mitigating concerns related to physical decline and psychosocial loss.

The present study was limited in several ways. First, the responses relating to information overload, cyberchondria, and self-perceptions of aging were subjective and susceptible to recall bias. The measurement of vaccine hesitancy relied on a limited number of items and may not capture all dimensions of the constructs. Second, the sample characteristics may limit the generalizability of our findings. The recruitment via online survey platform resulted in a sample primarily composed of active internet users, potentially excluding those with limited digital literacy or those in the oldest-old category (e.g., aged 80 and above). Furthermore, the predominance of male participants means sample may not fully represent the demographic structure of the broader Chinese older population. While this group of frequent internet users represents the primary users affected by cyberchondria and information overload, further studies should employ stratified sampling or offline recruitment to include a more diverse range of participants. Third, the cross-sectional design of this study limits the ability to draw definitive causal relationships, and the results should be strictly interpreted as associative relationships. Finally, while our model explained a significant portion of the variance in vaccine hesitancy, future studies could incorporate other factors, such as trust in medical professionals, prior vaccination experience, and social influence from family members, to provide a more comprehensive understanding of the topic. Similarly, more research is required to explore the moderating roles of socioeconomic characteristics, digital literacy, and health status of older internet users on the proposed pathways.

## Data Availability

The original contributions presented in the study are included in the article/supplementary material, further inquiries can be directed to the corresponding author.
